# Real-Time Imaging of Retinal Ganglion Cell Apoptosis

**DOI:** 10.3390/cells7060060

**Published:** 2018-06-15

**Authors:** Timothy E. Yap, Piero Donna, Melanie T. Almonte, Maria Francesca Cordeiro

**Affiliations:** 1 The Western Eye Hospital, Imperial College Healthcare NHS Trust (ICHNT), London NW1 5QH, UK; timothyedward.yap@nhs.net; 2 The Imperial College Ophthalmic Research Group (ICORG), Imperial College London, London NW1 5QH, UK; donnapiero@outlook.it (P.D.); melanie.almonte@nhs.net (M.T.A.); 3 Glaucoma and Retinal Neurodegeneration Group, Department of Visual Neuroscience, UCL Institute of Ophthalmology, London EC1V 9EL, UK

**Keywords:** retinal ganglion cell, apoptosis, neurodegeneration, glaucoma, annexin, imaging

## Abstract

Monitoring real-time apoptosis in-vivo is an unmet need of neurodegeneration science, both in clinical and research settings. For patients, earlier diagnosis before the onset of symptoms provides a window of time in which to instigate treatment. For researchers, being able to objectively monitor the rates of underlying degenerative processes at a cellular level provides a biomarker with which to test novel therapeutics. The DARC (Detection of Apoptosing Retinal Cells) project has developed a minimally invasive method using fluorescent annexin A5 to detect rates of apoptosis in retinal ganglion cells, the key pathological process in glaucoma. Numerous animal studies have used DARC to show efficacy of novel, pressure-independent treatment strategies in models of glaucoma and other conditions where retinal apoptosis is reported, including Alzheimer’s disease. This may forge exciting new links in the clinical science of treating both cognitive and visual decline. Human trials are now underway, successfully demonstrating the safety and efficacy of the technique to differentiate patients with progressive neurodegeneration from healthy individuals. We review the current perspectives on retinal ganglion cell apoptosis, the way in which this can be imaged, and the exciting advantages that these future methods hold in store.

## 1. The Cellular Basis of Glaucomatous Degeneration

### 1.1. Background to Glaucoma

Glaucoma is a progressive, sight-threatening neurodegenerative optic neuropathy thought to be predominantly characterized by apoptosis of retinal ganglion cells (RGCs) [1,2]. It is classically associated with loss of retinal nerve fiber layer and optic disc ‘cupping’, leading to characteristic mid-peripheral arcuate visual field defects [3]. It affects roughly 3.5% of the world’s population [4] and is rising in prevalence with increasingly ageing populations. Estimates suggest worldwide sufferers could total 111.8 million by the year 2040, demonstrating the necessity for more sophisticated ways to image this condition, especially at a cellular level. This is required in order to earlier identify those in need of treatment, and minimize visual loss. Novel biomarkers will also be key to researching new treatments that halt disease progression or even restore currently irreversible sight loss.

The pathogenesis of RGC loss in glaucoma is thought to be a complex interaction between genetic, structural and environmental influences [5]. The main risk factor and only current treatment target via medical, surgical and laser means is high intraocular pressure (IOP), whereby an imbalance between aqueous humour secretion from the ciliary body, and drainage via the trabecular meshwork and uveoscleral pathways occurs. Other risk factors include increasing age, family history, systemic and topical steroids, high myopia, black descent, and vascular dysregulation [5]. The most common subtype of glaucoma is primary open-angle glaucoma (POAG), to which the rate of conversion from simple ocular hypertension has been estimated at 9.5% of untreated patients, according to the ocular hypertension treatment trial (OHTT) during their five-year follow-up period. However after treatment, 4.4% still progressed to glaucomatous damage and vision loss [6], emphasizing the importance of understanding the contributing pressure-independent mechanisms and how to target them with treatment [7,8].

### 1.2. Visual Pathway in Glaucoma

The anatomical arrangement of the relevant visual pathway cell types has traditionally been the focus in understanding the decline in their function. Visual perception is first acquired by photoreceptors, which perform visual phototransduction to convert photons of light into electrical signals [9]. These specialized sensory neurons relay information to interneurons such as horizontal, bipolar and amacrine cells for further processing, the cell bodies of which constitute the inner nuclear layer [9]. These in turn relay processed visual information to the retinal ganglion cells, whose axons form the retinal nerve fiber layer, conveying signals to the brain via the optic nerve. These nerve fibers exit the globe via the lamina cribrosa, a mesh-like collagen structure that represents the weakest point of the sclera, where optic nerve fibers are purported to undergo mechanical stress; one of the several proposed triggers of retinal ganglion cell apoptosis [5]. This damage is thought to disrupt axonal transport from the brainstem, resulting in vesicle congestion and structural disarray in the peri-laminar fibers, as observed in several animal models of glaucoma and human post-mortem specimens [10,11]. It has also been observed that axonal degeneration is more extensive and closely correlated with increases in IOP when compared to RGC cell bodies, supporting this theory [12]. Nevertheless, the relationship with IOP is still not fully understood, given that disease frequency varies between certain races in the context of similar average IOPs [13].

### 1.3. Cellular Events in Glaucoma

Given that structural examination of the visual pathways has not fully explained glaucoma pathophysiology, attention has turned to intracellular events. ‘Programmed’ cell death of RGCs by apoptosis is thought to be the predominant cause of visual loss in glaucoma. Apoptosis was first described by Kerr et al. in 1972 [14] and is characterized by cellular shrinkage, membrane blebbing, and pyknotic nuclei with cleavage of nucleic acids and cytoskeletal proteins by endonucleases (caspases) and proteases [15]. The evidence for its role in glaucoma is largely due to the lack of necrotic processes seen in glaucomatous degeneration. Necrosis involves ATP depletion, resulting in the loss of membrane integrity, mitochondrial dysfunction and cell lysis, leading to inflammation. Whereas, nuclear changes in apoptosis are typically absent until later phases [16]. In contrast, a defining feature of apoptotic degeneration is disassembly of DNA into similarly-sized 180–200 base pair fragments [17], detected in raised levels in human glaucoma patients using methods such as the TUNEL (Terminal deoxynucleotidyl transferase (TdT) dUTP Nick-End Labeling) assay [1,2,18]. Pyroptosis is another proposed type of cell death containing features of both apoptosis and necrosis [19]. This is capase 1-dependent programmed cell death that is distinct to apoptosis in that it is a pro-inflammatory process that has been studied in microbial infections and myocardial infarction [20]. Given the role caspases play in pruning neurons during development, the involvement of this process in glaucomatous degeneration and as a possible treatment target has become a focus of interest [21].

**Figure 1:**
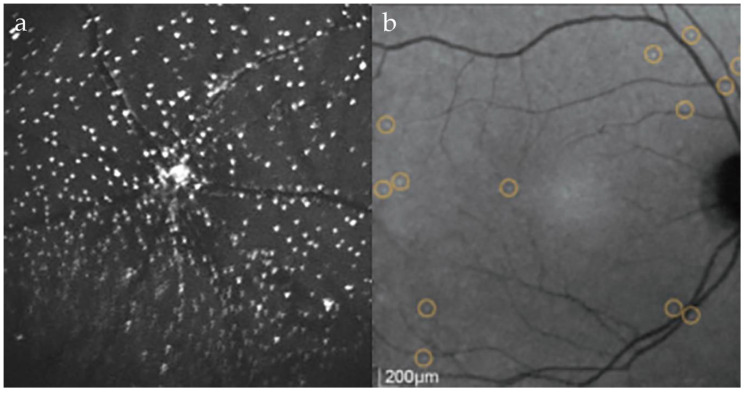
DARC imaging highlighting apoptosing retinal ganglion cells (**a**) using intravitreal ANX776 in a rat model of glaucoma following episcleral vein injections of hypertonic saline [39]; (**b**) using intravenous ANX776 in a human glaucoma patient shown to have progressive disease [40].

Several cellular processes have been suggested as triggers of accelerated RGC death (see Figure 1). One such concept is the alteration in the transport of brain-derived neurotrophic factor (BDNF) to RGC somata observed in rodent glaucoma models [22], along with evidence showing that intravitreal injections of BDNF can lower the rate of RGC loss [23]. However, knockout mice (bdnf −/−) have failed to exhibit reduced numbers of RGC axons, suggesting that this theory is not overwhelmingly comprehensive [24]. ‘Excitotoxicity’ is another major theory implicating excess glutamate transmission, the major excitatory neurotransmitter in the central nervous system. Some work has reported glutamate to be present at increased levels in the vitreous samples of glaucomatous human and animal eyes [25,26], although subsequent studies have failed to corroborate these findings [27,28,29]. The theory suggests that overstimulation of glutamate receptors (possibly in response to hypoxic injury [30]) leads to an apoptotic cascade of increased free radicals, increased intracellular calcium, and eventual caspase activation. To compound this theory, reduced glutamate transporters and therefore reduced clearance of extracellular glutamate has been reported [31]. Other proposed mechanisms have included mitochondrial dysfunction [32], oxidative stress [33], immunological factors [34,35], and vascular dysregulation [36]. Intracellular processes in other cell types that may lead to glaucomatous RGC death include mechanical stretching of lamina cribrosa cell membranes in which induction of calcium-induced calcium release from mitochondria has been demonstrated [37]. Similarly in the trabecular meshwork, protein misfolding and endoplasmic-reticulum stress may lead to increased IOP and glaucomatous degeneration [38].

## 2. Annexin A5 as a Marker of Cells Undergoing Apoptosis

### 2.1. Apoptosis

The dysregulation of apoptosis has been implicated in a plethora of conditions, both in terms of acceleration in conditions such as Alzheimer’s [41], Huntingdon’s [42] and Parkinson’s disease (PD) [43], and resistance to apoptosis found in several forms of malignancy [44,45]. This has naturally led to the development of various methods by which to detect apoptosis, both in- and ex-vivo [46] in order to clinically diagnose and monitor these conditions, and also as a potential surrogate marker to prove the efficacy of novel treatments. The main advantage of being able to monitor cells in-vivo as opposed to previously used methods such as TUNEL labeling [18] is the ability to perform repeated examinations on the same subjects to monitor individual cells over time.

### 2.2. Annexin and Apoptosis

Annexin A5 is a 36kDa endogenous protein ubiquitously expressed in animals and humans. Its biological function is unclear but is thought to involve membrane permeability and repair [47], autophagy [48], modulation of protein kinase C and phospholipase A activity [49], as well as anti-endotoxin [50] and anti-thrombotic [51] activities. Annexin A5 has a high, calcium-dependent electrostatic affinity for phosphatidylserine (PS), an anionic phospholipid displayed on the outer leaflet of cell membranes early in apoptosis [52]. PS is normally maintained in an asymmetric distribution across the cell membrane by ATP-dependent ‘flippases’, in favor of the inner, cytosolic leaflet [53,54,55]. However, during early apoptosis it is displayed on the outer leaflet of the cell membrane, acting as an ‘eat-me’ signal to attract phagocytes [56,57]. This is through a combination of downregulation of such flippases, and activation of scramblase proteins that allow exposure of PS on the outer cell surface by calcium-dependent movement of phospholipids across the membrane leaflets [58].

### 2.3. Uses of Annexin

The use of annexin A5 was developed as an in-vitro method of detecting apoptosis, favored for its high degree of sensitivity [59] and often conjugated with vital dyes such as 7-amino-acitinomysin (7-AAD) or propidium iodide (PI). Human studies using annexin A5 to measure levels of apoptosis have used radiolabels such as ^8^F-, ^124^I or ^99m^Tc in combination with positron emission tomography (PET) and single-photon emission computed tomography (SPECT) imaging to characterize apoptosis in opaque tissues such as in studies of myocardial infarction [60,61], cardiac allograft rejection [62], cardiac tumours [63], haematological malignancies [64,65], head and neck carcinoma [66], stroke medicine [67] and inflammatory bowel disease [68]. However, the deep positioning and opaque nature of many of the organs limits the precision with which tracer signals can be detected.

### 2.4. Other Methods for Imaging Apoptosis

In addition to annexin A5, other techniques have been described to image in-vivo apoptosis, targeting changes in cell membrane permeability, cell surface markers and caspase-dependent cell death. 18F-labelled NST-732 (ApoTrace^®^) is one such previously available marker for use in conjunction with PET imaging that has been shown to exclusively permeate membranes of dying cells early in the process of cellular death, preceding complete loss of membrane integrity [69]. This was shown in animal models of lymphoma, renal ischemia and cerebral infarction. Z-DEVD-aminoluciferin is another apoptosis marker used in-vivo to detect apoptosis with luminescence imaging, enabled through cleavage by caspase-3 in apoptotic cells [70]. Active caspase labelling can also be achieved using fluorescently-labelled poly-caspase inhibitors (FLIVO^®^, Neuromics, Minneapolis, MN, USA) and have has been used to study tumors in animal models by forming covalent bonds to intracellular caspases of apoptosing cells [71].

## 3. Single-Cell Resolution Imaging of the Retina

### 3.1. Imaging the Eye

The optically transparent nature of the cornea and lens permits the unique opportunity to image the retina at a cellular resolution in the eye. The steps towards achieving this goal have included the development of scanning laser ophthalmoscopy (SLO) in the 1980s [72], optical coherence tomography (OCT) in the 1990s [73], and most recently, adaptive optics (AO) technology to enhance both these modalities (AO-SLO and AO-OCT) to achieve transverse and axial resolutions of up to 2–3 µm [74,75].

### 3.2. Retinal Cell Imaging

En-face imaging of photoreceptors with AO visualizes a regular arrangement of well-contrasted cones in a mosaic pattern [76], enabling determination of cone density and receptor type (S, M, L cones) [77]. However, imaging of rods and the retinal pigment epithelium (RPE) has proven more challenging. Rods, due to their small size (2 μm diameter) and angled positioning, and RPE due to the low intrinsic contrast of cells underlying the photoreceptor layer, which additionally acts to scatter the light. For this reason, RPE imaging was initially most successful in eyes with overlying photoreceptor loss [78]. ‘Dark field’ imaging has been subsequently developed using modified AO-SLO with a larger aperture and a central filament blocking back scattered light in order to depict the characteristic hexagonal RPE morphology [79]. AO techniques have also enabled imaging of blood vessel mural cells [80] and erythrocytes [81]. Linking structure to function using this method, ‘ill’ cone photoreceptors have been observed to display different reflectance patterns to healthy cones in a rod-cone dystrophy [82]. AO-enhanced techniques have also been used to study other diseases causing photoreceptor disturbance, including age-related macular degeneration (AMD) [83], macular telangiectasia type 2 [84] and hydroxychloroquine toxicity [85]. Investigation of inflammatory conditions has advanced with AO, including imaging of photoreceptor disruption in white dot syndromes [86] and retinal vasculitis [87]. Factors limiting the widespread uptake of AO include the slow acquisition speed that demands maximal patient cooperation, the small field of view, and the dramatic impact media opacities have on image quality. Furthermore, the near-transparent nature of the inner retina, required for light to reach the photoreceptors, has left us bereft of effective ways to image certain cell types. These include subtypes of intermediate neurons, and most critically for glaucoma, retinal ganglion cells.

## 4. Imaging Retinal Ganglion Cells

### 4.1. The Challenge of Imaging Retinal Ganglion Cells

Retinal ganglion cells are challenging to image due to their low contrast edges [88]. Although widely used in glaucoma management, OCT technology is only able to measure the combined thickness of RGC axons (as the RNFL thickness). Even though RNFL thickness is thought to be a sensitive indicator of early disease, tens of thousands of individual nerve fibers may be lost prior to a trend in RNFL thickness being detected [89]. Reduced reflectance of the RNFL has been reported to precede thinning [90]; however this measure has not infiltrated clinical practice. AO imaging has shown the variation in nerve fiber layer health for a given RNFL thickness on OCT, indicating that higher resolution imaging has the potential to provide new levels of insight into the demise of RGCs [91]. AO techniques have recently been combined with two-photon excitation fluorescence (TPF-AOSLO) to harness the autofluorescence of endogenous fluorophores in the retina to image individual RGC soma, be this in a very small 100 μm × 100 μm window of retina. Whilst undoubtedly useful in further exploring real-time morphological changes in animal models, this technique currently has limited use in humans due to the high light levels required [92].

### 4.2. DARC Technology

By using fluorescently-labelled annexin A5, the DARC (Detection of Apoptosing Retinal Cells) technique has made headway in imaging real-time functionally-relevant information by achieving the labeling of apoptosing retinal ganglion cells in vivo. Two fluorescently-labelled annexin A5 molecules have been trialed. The first, Alexa Fluor 488-labeled annexin A5 with excitation/emission wavelengths of 495/519 nm. The second, ANX776, is a fluorescently-labelled variant of human annexin A5, RhAnnexin V128. Using this variant molecule enabled a covalent bond between its cysteine residue and the maleimide form of the fluorescent dye, Dy776-maleimide (Dy-776-mal) [40]. ANX776 has been developed with excitation/emission wavelengths in a similar near-infrared range (771/793 nm) to indocyanine green (ICG), currently used to investigate chorioretinal conditions such as idiopathic polypoidal choroidal vasculopathy (IPCV) [93,94].

DARC imaging uses modified confocal scanning laser ophthalmoscopy (cSLO) (Heidelberg Retina Angiograph 2, Heidelberg Engineering, Dossenheim, Germany) [95,96]. cSLO provides high-contrast retinal images by raster scan illumination using a spot laser, and the passing of returning light through a confocal pinhole to select the focal volume of interest, minimizing optical cross-talk and enhancing axial resolution [97]. ICG angiography settings of 786nm diode laser illumination are used, with an 800 nm barrier filter on the photodetector to highlight uptake of ANX776 to the membrane of apoptosing cells. In order to achieve the highest signal-to-noise ratio possible, the manufacturer’s eye tracking technology and averaging of 100 frames are used. Images acquired so far pertain to a field of view of between 30 and 55° and can be centered at the fovea or the optic disc. Compensating for other non-enhancing structures in the eye and non-linear distortions is achieved by post-acquisition transformations using techniques described elsewhere [98,99,100]. Real-time quantification of RGC apoptosis is then acquired by detecting the individual DARC spots, seen as 12–16 μm hyperfluorescent points, using a template-matching approach resulting in a ‘DARC count’ (see Figure 1) [101].

### 4.3. Experimental Studies with DARC

DARC has been used in multiple animal studies to monitor rates of in-vivo retinal apoptosis, both in order to study the natural history of neurodegeneration in relation to glaucoma and other conditions, and often hand-in-hand with trials of neuroprotective treatment strategies (see Table 1). The well-documented rodent glaucoma models have been induced chemically by episcleral vein injections of hypertonic saline to induce chronic ocular hypertension [102], and surgically with partial optic nerve transection (pONT) in order to observe primary and secondary degeneration of RGCs [103]. A model using intravitreal injections of staurosporine, a non-selective protein kinase inhibitor, has also been developed for use with DARC to induce rapid and extensive RGC apoptosis [104,105]. Others used with DARC include an intraperitoneal rotenone-induced model of Parkinson’s disease [106], and a transgenic murine model of diabetes, C57BL/6-Ins2^Akita^/J [107].

RGC apoptosis was first successfully imaged in real-time, in-vivo with DARC in 2004 in a proof-of-concept study, examining the accuracy and sensitivity of this novel technique [105]. Verification of the DARC spots as apoptosing retinal ganglion cells was shown using co-labelling of the structures of interest. RGCs were stained using retrograde injections of DiAsp, 4-(4-(didecylamino)styryl)-*N*-methylpyridinium (4-Di-10-Asp, Invitrogen™ Molecular Probes™, Fisher Scientific, Waltham, MA, USA) to rat superior colliculi [121], and apoptosing cells dual-labelled with annexin A5 and Cy5-labeled anti-caspase-3 antibodies. These results showed a good correlation between the staining patterns [105]. Further work has gone on to characterize the pathogenesis of glaucomatous retinal neurodegeneration using this technique, confirming the significant correlation between RGC apoptosis with magnitude of IOP elevation, in addition to implicating effects on the extra-cellular matrix [108]. In transgenic diabetic mice, there was found a significantly increased rate of RGC apoptosis at eight weeks of age, suggesting DARC may be useful in the detection of early diabetic retinopathy [107]. Amyloid-beta, implicated in Alzheimer’s disease pathogenesis, has been shown to co-localize with apoptosing RGCs in glaucoma models, whilst also inducing apoptosis in a time and dose-dependent manner [110]. This revealed an important pathogenic crossover between these two conditions, with possible future clinical implications for the spectrum of age-related neurodegeneration. Finally, DARC has demonstrated imaging of both inner and outer nuclear layer cell death in vivo [111], which also may permit the identification of photoreceptor apoptosis in dry age-related macular degeneration [114].

### 4.4. DARC as an Outcome Measure

Pre-clinical studies have shown neuroprotective effects of several existing drugs. Through DARC, the systemic administration of liposome-encapsulated rosiglitazone in an animal model of PD was shown to have a neuroprotective effect on the retina and central nervous system [118]. In addition, DARC studies have provided new support for the IOP-independent neuroprotective mechanism of the alpha-2 adrenergic receptor agonists (*α*2ARAs) brimonidine and clonidine via the amyloid-beta (A*β*) and secreted amyloid precursor protein *α* (sAPP*α*) pathways [119]. When applied topically, memantine (an NMDA receptor antagonist used in Alzheimer’s disease) loaded onto PLGA-PEG nanoparticles (MEM-NP) significantly reduced RGC loss [120]. A similar neuroprotective effect was seen with topical coenzyme Q10 by assessing RGCs in vivo using DARC [39].

**Figure 2:**
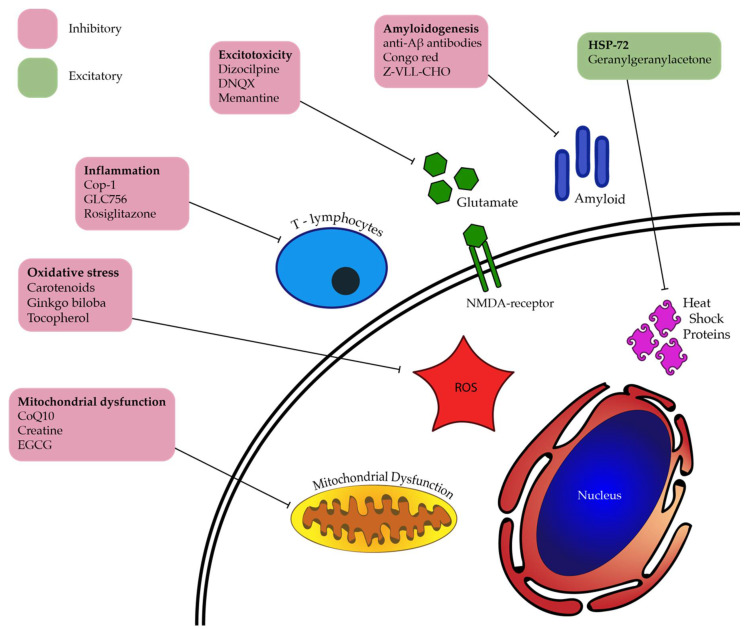
Potential RGC-neuroprotective agents and their targeted pathogenic processes, some of which have been studied with DARC imaging. (ROS: Reactive Oxygen Species).

DARC has also allowed for the effect of novel agents to be evaluated. Targeting glutamate excitotoxicity, the broad-spectrum NMDA receptor antagonist MK801 has been shown to be a more effective neuroprotector of RGCs compared to NR2B-selective NMDA receptor antagonist ifenprodil, especially when combined with group II metabotropic glutamate receptor (mGluR) agonist LY354740 [109]. Targeting amyloidogenic effects, the amyloid-beta aggregation modulator MRZ-99030 provided dose-dependent reduction in RGC apoptosis seen in the Morrison rat ocular hypertension model of glaucoma [115]. 2-Cl-IB-MECA, a selective adenosine A3 receptor (A_3_R) agonist impeded RGC apoptosis demonstrated via three possible mechanisms—glutamate excitotoxicity, ischaemia-reperfusion injury, and axonal damage observed in a pONT model [117]; however other studies examining this mechanism of action at varying concentrations and with differing cell types have had conflicting results [122]. A successful cell-based therapy has also been developed using DARC, involving a novel method of Schwann cell delivery via direct optic nerve sheath (DONS) application. DARC was used to image the pONT model used, demonstrating RGC protection by targeting secondary degeneration [116]. This study demonstrated the unique potential of DARC to act as a surrogate in repeatedly assessing the effects of treatment on RGC apoptosis over time in the same living subjects, without specifically detecting the uptake of Schwann cells directly. A diagrammatic summary of these and other candidates that may be useful in treating retinal neurodegeneration is displayed in Figure 2.

## 5. The Use of DARC Imaging in Humans

### 5.1. Phase 1 DARC Study

Following on from the success of animal studies, DARC imaging has now been trialled in human subjects to provide the first demonstration of in-vivo individual retinal cell apoptosis visualization in the human retina. The proof-of-concept Phase I clinical trial comparing eight progressing glaucoma patients with eight healthy volunteers was carried out to demonstrate the safety and efficacy of the technique [40]. Using a range of intravenous ANX776 doses given to four patient groups (0.1, 0.2, 0.4 and 0.5 mg), a significant increase in DARC count was observed in glaucomatous subjects (2.37-fold, 95% confidence interval: 1.4–4.03, *p* < 0.005), an effect especially evident at the 0.4mg dose where mean DARC count increased from 10 to 25 (*n* = 4, *p* <0.005). DARC count was also found to correlate with increased cup-disc-ratio, and reduced central corneal thickness (R = −0.68, *p* = 0.006), previously controversially cited as an independent risk factor for glaucoma [123,124]. Additional post-hoc analysis of the data also revealed a significant association between DARC count and increasing rates of glaucomatous progression (Dunn’s multiple comparison test, *p* < 0.05), however not compared with patients with more stable rates of progression, bearing in mind the small sample size.

There were no serious adverse events recorded during the study. All minor adverse events were considered unrelated to the ANX776, and included metatarsal inflammation, transient dizziness and headache in patients who had experienced these symptoms prior to the study, one case of influenza, and two reports related to the phlebotomy itself, namely a haematoma and discomfort. ANX776 pharmacokinetics were monitored in all subjects, demonstrating fast absorption (T_max_, time to maximum concentration = 5.0–7.0 min), dose-dependent mean maximum serum concentrations (range: 5.5 to 40.9 ng/mL), short half-life (range 36.4 to 20 min for the 0.1 to 0.5 mg doses, respectively), and no accumulation (minimum serum concentration 0.6–1.0 ng/mL).

### 5.2. DARC as a Surrogate for Neurodegeneration

Thus far, studies using DARC have extensively demonstrated its utility in examining the characteristics and pathogenesis of RGC neurodegeneration, and the potential for researching neuroprotective treatment strategies, including its safe use in humans. Not only is this advantageous in the study of the natural history and treatment of glaucoma, but holds potential uses in other neurodegenerative conditions such as Alzheimer’s and Parkinson’s disease, whereby the eye may prove a useful ‘window’ through which to investigate novel treatments and improve early diagnosis, using DARC. Clinically, DARC holds potential in establishing baseline disease activity, monitoring treatment efficacy, and investigating those patients in whom other methods have fallen short.

### 5.3. Potential of DARC in Glaucoma Diagnosis

The current gold-standard method for diagnosis and monitoring of glaucoma in the clinical setting is standard automated perimetry (SAP). SAP involves automated visual field testing using protocols such as those developed by the SITA (Swedish Interactive Thresholding Algorithm) group in order to provide reproducible visual field plots in a time period acceptable to most patients [125,126]. However, this method of investigation relies on the patient’s ability to carry out the test, which involves holding their head in a certain position for several minutes, keeping concentration on a fixation target, and is non-specific for any particular cause of visual deterioration, be it glaucoma, cataract or macular pathology, although the characteristics of field defects commonly differ between these pathologies. Often the elderly, those lacking concentration and comprehension, or physical agility, will be penalized due to their lack of ability to carry out the test with appropriate reliability indices, estimated at more than a third of tests [127]. It has been shown that even the average able person undergoes a certain ‘learning curve’ during their first few attempts that may counteract evidence of early disease progression [128]. Furthermore, recent evidence suggests that the widely used SITA 24-2 protocol commonly misses early disease due to the low density of testing points in the central 10 degrees [129]. Conversely, visual fields in advanced glaucoma also provide challenges of reproducibility due to difficulties with fixation and extensive field loss. From the clinician’s standpoint, accurate interpretation of the results can often be subjective, requiring the expertise of an experienced interpreter. Once a visual field defect is detected, progression analysis over time is required to determine disease activity and the intervention required. For example, some researchers have proposed that four-monthly testing is required over two years to reliably detect a change of −4 dB [130], a target not commonly applied to most patients. The United Kingdom Glaucoma Treatment Study (UKGTS) in 2015 was the first randomized controlled trial to directly demonstrate visual field preservation with an IOP-lowering therapy [131]. This was achieved over 24 months, a timeframe that is encouraging for visual field progression as an endpoint in future clinical trials. Given that the ‘pre-perimetric’ period of glaucoma prior to visual field defects occurring is thought to represent a loss of 30% of RGCs over an estimated 2–8 years [132], new ways to anticipate field loss are required to avoid waiting for unnecessary, as yet irreversible, visual loss to occur in order to confirm clinical suspicion.

### 5.4. Current Outcome Measures in Glaucoma

The majority of current treatments have been proven on the basis of reduced IOP as a presumed surrogate marker for progression of visual field loss. As a consequence, IOP remains the only treatment target in glaucoma management. The large scale-trials demonstrating association between these variables are those such as the Ocular Hypertension Treatment Trial (OHTT) [6] and the Early Manifest Glaucoma Trial (EMGT) [133]. Equally, many clinical decisions are made on the basis of IOP measured in consultations. It is therefore important to remember the effects of diurnal variation [134] and central corneal thickness [135] on IOP measurement, especially in the context of a single, one-off measurement, along with other postural factors that are more difficult to control [136,137,138]. Moreover, even taking multiple readings throughout the day (phasing) to characterize an individual’s IOP fluctuation has been shown to lack repeatability [139]. IOP as an effective surrogate marker also comes into question when considering several groups of patients. These include those patients who are at risk of under-treatment in whom glaucomatous progression occurs in the absence of high IOP (normal-tension glaucoma), and those who progress even on adequate anti-ocular hypertensive treatment. Secondly, there is the patient group who may be over-treated based on their intraocular pressure alone.

Recent advances in imaging technology have provided biomarkers with which to detect earlier glaucomatous disease activity. Optical coherence tomography (OCT) is now widely used in the routine assessment of glaucoma patients [140]. This provides non-invasive cross-sectional imaging of the optic nerve head and macula, able to monitor structural parameters of interest in glaucoma such as retinal nerve fiber layer (RNFL) and ganglion cell complex thickness, along with more traditionally observed markers such as optic nerve head rim area and cup-to-disc ratio. Variability of ‘normal’ optic disc appearance creates difficulty when attempting to diagnose glaucoma on initial assessment. This can be present in cases such as myopia, varying optic disc size, tilted discs and congenital abnormalities, in addition to optic neuropathies of non-glaucomatous etiology [141,142,143]. Baseline imaging parameters have been found to hold moderate ability to predict future glaucomatous progression as per the Advanced Imaging for Glaucoma Study (Ganglion Cell Complex Focal Loss Volume, GCC-FLV: Area under receiver operating characteristic (AUROC) curve = 0.632). With progression analysis over time, more impressive predictive values set against the EMGT visual field criteria have been quoted (RNFL thinning in a composite model: HR = 8.44, 95% CI: 3.30–21.61) [144]. Similar to visual field assessment, awaiting loss of RNFL fibers to confirm diagnostic suspicion permits irreversible loss of visual field. 

### 5.5. DARC as an Exploratory Outcome Measure in Glaucoma

**Figure 3:**
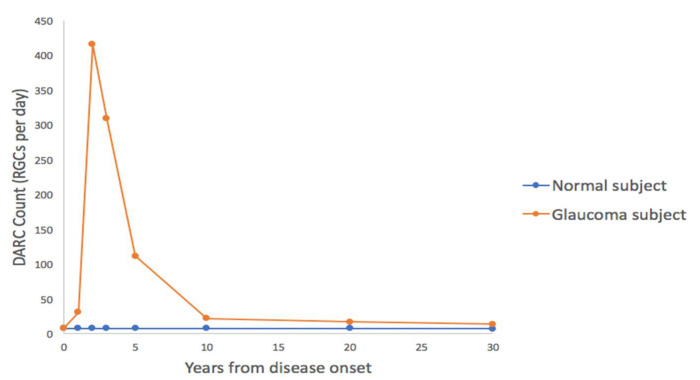
Lifespan-adjusted projection of DARC counts extrapolated from a rodent model of glaucoma, and superimposed onto a human disease course over 30 years. This demonstrates potential for diagnosis of early disease using DARC.

In comparison to these methods, DARC holds potential as a predictor of future visual field progression from a single baseline measurement [40], thus allowing for earlier treatment and preservation of vision. Furthermore, it has already been demonstrated as a powerful research tool by which to monitor in-vivo individual retinal cell apoptosis in models of both glaucoma and other neurodegenerative conditions (see Table 1). DARC may provide more universal and repeatable data in comparison to visual field testing, not relying on the patient’s ability to comply with testing procedures. In comparison with imaging, apoptosis rates in age-matched healthy subjects may provide a normal reference value against which it is easier to compare glaucoma suspects. It is the hope that patients with pre-perimetric glaucoma and subtle corresponding changes in imaging parameters may be afforded earlier diagnosis, in addition to those advanced glaucoma patients in whom having few remaining RGCs makes detecting nerve fiber loss increasingly difficult [145]. The key to the successful uptake of this technology will lie in the ability to differentiate absolute DARC counts between normal, stable and progressing patients. This question has begun to be addressed using a rat model of ocular hypertension, extrapolating data onto a human lifespan to estimate expected DARC counts following a hypertensive insult. This model used a healthy baseline of 0.3% annual RGC loss, equating to a DARC count of 8 per day. This is substantially lower than the peak projected RGC loss at two years from onset, which is estimated at 416 RGCs per day (15.38% annual RGC loss) (Figure 3) [146]. When compared to human data from Phase I, it was shown an average glaucomatous RGC loss of 4% [147] correlated with the DARC counts found in the 0.4mg treatment group [40].

## 6. DARC—Next Steps

Results from Phase II of the DARC study are due to be reported soon. The aim of this study was to assess DARC in up to 120 patients, including healthy volunteers and those with glaucoma, optic neuritis, age-related macular degeneration, and patients with Down Syndrome as a model of Alzheimer’s disease (under strict ethical considerations). In addition to defining the range of DARC counts in humans, health and disease, this study will also address if it is possible to differentiate distinct pathologies. Further work to improve the technique involves establishing a larger normal and glaucoma patient dataset to fully characterize the thresholds in DARC counts between different stages of the disease. 

Whilst research into alternative routes of administration of the fluorescent marker will continue, the technique in its current form would foreseeably continue to be a valuable biomarker or even surrogate endpoint in the investigation of neuroprotective treatment strategies. For clinical use in the current form, it would be a valuable tool in measuring the response to treatment, although it may also have a role in providing baseline recordings, aiding in cases of diagnostic uncertainty, determining justification for management decisions, or for use in those patients in whom there has been failure of alternative investigations. However, intravenous injections at every follow-up visit is unlikely to be practically feasible or favored by patients. The procedure must also be performed in a hospital or clinic setting by a trained clinician due to the invasive elements of the procedure. In addition to initial discomfort, inserting an intravenous catheter also requires a trained technician and carries with it the small risk of local and systemic infection. A non-invasive method of administration is being developed at this time. In the Phase II study, patients had to undergo three scans at 15 min, 2 h and 4 h to select the best time point for imaging. The results will soon be available, as this will impact clinical time needed for the investigation. DARC also has an inability to detect the number of remaining retinal cells. This emphasizes the fact that the placing of DARC in the immediate future is likely to be as one of an armory of diagnostic tools available, used in combination with other established and novel investigations that can provide accompanying information, offering the researcher and clinician the greatest choice of tools with which to halt and hopefully cure glaucoma, and other neurodegenerative conditions. In the longer-term, DARC can only be validated in trials where it is compared to conventional clinical endpoints; however, it could be used as an exploratory outcome measure until then.

## Figures and Tables

**Table 1:** Studies using DARC imaging.

Focus of Study	Model	Finding	Reference
Proof of concept	Rat	First retinal cell apoptosis imaging with DARC in vivo. Histological validation of the DARC technique confirms apoptosing RGCs.	[105]
IOP *(Pathogenesis)*	Rat	RGC apoptosis is strongly correlated with elevated IOP, and changes to the extra-cellular matrix induced by raised IOP.	[108]
NMDA receptor antagonism *(Treatment)*	Rat	Demonstration of a staurosporine-induced rat ocular hypertension model in testing neuroprotective strategies. Broad-spectrum NMDA receptor antagonist MK801 is a more effective neuroprotector than NR2B-selective NMDA receptor antagonist ifenprodil, especially when combined with group II mGluR agonist LY354740.	[109]
Beta-amyloid *(Pathogenesis)*	Rat	Beta-amyloid, implicated in Alzheimer’s disease, co-localizes with apoptosing retinal ganglion cells, and induces RGC apoptosis in a time and dose-dependent manner.	[110]
Diabetic retinopathy *(Pathogenesis)*	Mouse	RGC apoptosis was significantly higher in transgenic diabetic mice at eight weeks of age when compared to normal controls, suggesting DARC may be useful in early detection of diabetic retinopathy.	[107]
Laser exposure *(Pathogenesis)*	Rat	First use of DARC to image inner nuclear layer apoptosis after laser treatment with frequency-doubled Nd:YAG retinal laser. Increasing duration and power of laser led to more inner retinal layer involvement, with dose-dependent correlation of laser exposure and DARC spot density, along with lesion area and elevation.	[111]
Light damage *(Pathogenesis)*	Rat	In vivo demonstration of outer nuclear layer apoptosis in response to blue light exposure. Histological analysis confirmed photoreceptor death.	[112]
Proof of concept	Rat	Spectrally distinct fluorescent markers were used to monitor both early and late apoptosis and necrosis in individual cells, in real-time.	[113]
Dry AMD *(Pathogenesis)*	Mouse	Identification of photoreceptor apoptosis in dry age-related macular degeneration (AMD).	[114]
Amyloid-beta *(Treatment)*	Rat	A dose-dependent neuroprotective effect from systemic injections of the amyloid-beta aggregation modulator MRZ-99030.	[115]
DONS *(Treatment)*	Rat	A novel method of direct optic nerve sheath (DONS) delivery of Schwann cells in a partial optic nerve transection model of secondary degeneration is protective against RGC apoptosis, compared to intravitreal delivery.	[116]
Adenosine A3 agonists *(Treatment)*	Rat	2-Cl-IB-MECA, a selective adenosine A3 agonist, is neuroprotective in vitro and in vivo.	[117]
Rosiglitazone *(Treatment)*	Rat	DARC used to demonstrate retinal changes in a rodent model of Parkinson’s disease. An enhanced neuroprotective effect against rotenone-induced damage was seen with liposome-encapsulated rosiglitazone.	[118]
Brimonidine *(Treatment)*	Rat	IOP-independent neuroprotective effect of alpha2 adrenergic receptor agonists (*α*2ARAs) brimonidine and clonidine.	[119]
Coenzyme Q10 *(Treatment)*	Rat	Topical coenzyme Q10 has a significant neuroprotective effect in a surgically-induced ocular hypertension model of glaucoma.	[39]
Proof of concept	Human	Intravenous ANX776 is a safe way to monitor rates of RGC apoptosis in humans using DARC imaging. A significant difference in DARC count was seen between progressing glaucoma patients and healthy controls.	[40]
Memantine *(Treatment)*	Rat	Memantine is an NMDA receptor antagonist, used in the treatment of Alzheimer’s disease. Topical memantine-loaded PLGA-PEG nanoparticles significantly reduced RGC loss in an experimental glaucoma model.	[120]
