# Correction: Yap et al. Real-Time Imaging of Retinal Ganglion Cell Apoptosis. *Cells* 2018, *7,* 60

**DOI:** 10.3390/cells14211690

**Published:** 2025-10-28

**Authors:** Timothy E. Yap, Piero Donna, Melanie T. Almonte, Maria Francesca Cordeiro

**Affiliations:** 1 The Western Eye Hospital, Imperial College Healthcare NHS Trust (ICHNT), London NW1 5QH, UK; 2 The Imperial College Ophthalmic Research Group (ICORG), Imperial College London, London NW1 5QH, UK; 3 Glaucoma and Retinal Neurodegeneration Group, Department of Visual Neuroscience, UCL Institute of Ophthalmology, London EC1V 9EL, UK

Missing Citation

In the original review publication [1], the original research publications first publishing the images displayed in Figure 1 were not cited in the legend. Citations for the relevant publications referenced elsewhere in the manuscript have now been inserted into the legend for Figure 1 and should read:

“Figure 1. DARC imaging highlighting apoptosing retinal ganglion cells (**a**) using intravitreal ANX776 in a rat model of glaucoma following episcleral vein injections of hypertonic saline [39]; (**b**) using intravenous ANX776 in a human glaucoma patient shown to have progressive disease [40].”

As the addition of references to Figure 1 caption caused the references to no longer appear in numerical order, the numbering of the references was reorganized after the caption of Figure 1.

The authors state that the scientific conclusions are unaffected. This correction was approved by the Academic Editor. The original publication has also been updated.
